# Experimental data on the properties of pelletization of palm kernel shell using sago starch and sodium acetate

**DOI:** 10.1016/j.dib.2020.106535

**Published:** 2020-11-15

**Authors:** Siti Abdul Halim, Nurul Razali, Noor Mohd

**Affiliations:** a Chemical Engineering Section, Malaysian Institute of Chemical and Bioengineering Technology (MICET), Universiti Kuala Lumpur, Lot 1988, Kawasan Perindustrian Bandar Vendor, Taboh Naning, 78000 Alor Gajah, Melaka, Malaysia; b Faculty of Ocean Engineering Technology and Informatic, Universiti Malaysia Terengganu, 21300 Kuala Terengganu, Terengganu, Malaysia

**Keywords:** Palm kernel shell, Pelletization, Binding agent, Sago starch, Sodium acetate

## Abstract

Pellet mixed with 5 wt% and 10 wt% of binders was tested. The pelleting process was performed using a pellet mill operated at 100 °C and at 50 MPa. The physical and chemical characteristics including hardness, high heating value and proximate analysis of pellet produces were obtained using durometer and through thermographic analysis and the results were reported in this paper. Bulk and unit density were determined according to ASABE standard. The dataset presented here are the data of palm kernel shell pellet prepared using two types of binder; (1) sago starch and (2) sodium acetate. The pelletization of palm kernel shell aimed to increase the density and strength of the palm kernel shell pellet and consequently provide better thermal degradation characteristics.

## Specifications Table

**Table:** 

Subject	Chemical Engineering
Specific subject area	Sustainability and Environment
Type of data	Table Figure
How data were acquired	Thermographic Analysis Durometer
Data format	Raw Calculated Analyzed
Parameters for data collection	Bulk density Unit density Hardness High heating value Ash content Moisture content Fixed carbon Volatile matter
Description of data collection	Palm kernel shell pellet was prepared at different composition of the binders. The data were collected through several analyses such as proximate analysis, bulk and unit density test, hardness test and thermal gravimetric analysis
Data source location	Institution: Malaysian Institute of Chemical and Bioengineering Technology (MICET), City/Town/Region: Taboh Naning, Alor Gajah Country: Malaysia
Data accessibility	All data are with this article

## Value of the Data

• The data are useful in comparing the impact of the addition of binding agents on physical and chemical properties of the pallet.
• The use of binding agent in pellets helps to increase the environmental and economic sustainability of biomass pellets which will further lead to a greater market share and boost the competitiveness of pellet producers. The data will then be a guidance for binder selection and application when compacting raw biomass into pellet with enhanced physical and chemical characteristics.
• The data set reported in this article therefore can be the baseline for pellet production from an extended raw material consisting of wide variety of fibrous residues from agriculture.

## 1 Data Description

Indonesia is the largest palm oil exporter followed by Malaysia, with 55.5% and 29% respectively [1]. There is a total of 5.8 million hectares of land under palm oil cultivation in Malaysia, where 19.5 and 2.5 million tons of crude palm oil and palm kernel cake have been produced respectively in 2018 [1]. These palm kernel shell (PKS) can be used as energy source for combined heat and power generation [2]. Pellets produces from biomass have the possibility to replace the use of coal or fossil fuel in co-firing heating in industrial operation and consequently reduces the energy cost. In addition, these pellets can also be used in power plants to solve insufficient power supply problem [3]. This article reports on how the addition of binder affects the strength and properties of the pellets [4] and in this case, two types of binder which are sodium acetate (SA) and sago starch (SS) were employed as shown in Table 1.

**Table 1:** Ratios of PKS material and binder to be pelletized.

Ratio of raw material and binder	Composition percentage (%) by weight		
	PKS	SA	SS
PKS	100	–	–
PKS + SA (90:10)	90	10	–
PKS + SS (90:10)	90	–	10
PKS + SA (95:5)	95	5	–
PKS + SS (95:5)	95	–	5
PKS + SA + SS (90:5:5)	90	5	5
PKS + SA + SS (80:10:10)	800	10	10

**Fig. 1:**
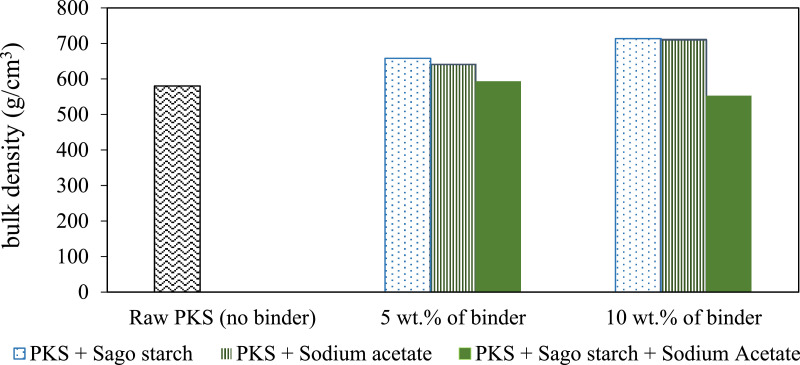
Bulk density for palm kernel shell pellet at different binder levels.

**Fig. 2:**
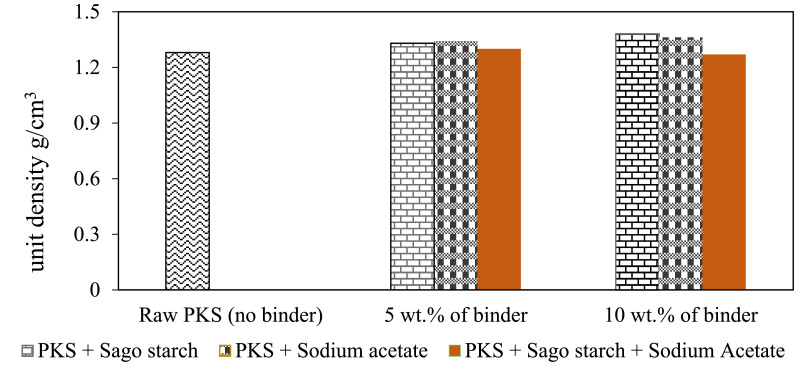
Graph of unit density for palm kernel shell pellet at different binder levels.

**Fig. 3:**
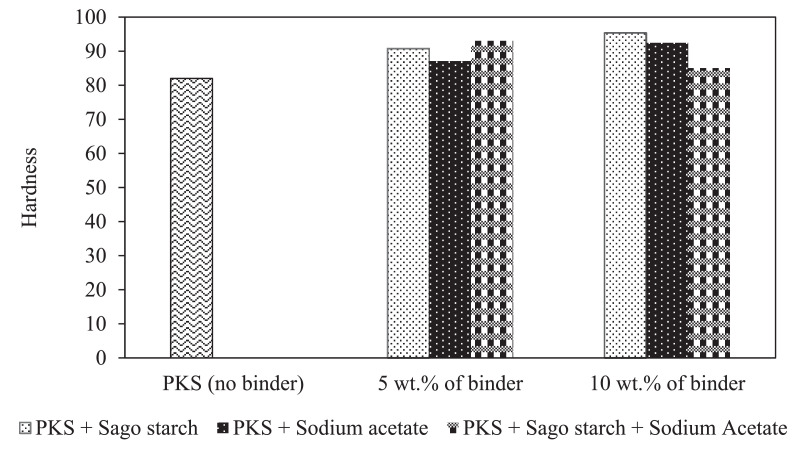
Hardness of PKS pellets at different binder level.

Table 2 shows the physical properties of palm kernel shell pellet mixed with different binder composition where the density and the hardness of the pellet were calculated (with standard deviation). Proximate analysis from different ratios of binder with PKS pellets were summarized in Table 3; where high heating values were measured using correlation proposed by Parikh and co-worker [5]. Figs. 1–3 show the comparison of bulk density, unit density and hardness between the PKS pellet and the pellet that has been mixed with SS and SA. Hardness measures a material's resistance to surface deformation.

**Table 2:** Physical properties of palm kernel shell pellet at different binder levels (mean ± standard deviation).

	Pellet properties						
Pellet ratio	Length (cm)	Diameter (cm)	Volume (cm^3^)	Mass (g)	Bulk density(kg/m^3^)	Unit density(kg/m^3^)	Hardness
Raw PKS (100:0)	2.71 ± 0.17	0.60	0.77 ± 0.04	0.98 ± 0.04	580.16 ± 1.71	1280 ± 0.02	82.0 ± 3.05
PKS + SS (95:5)	2.59 ± 0.17	0.60	0.73 ± 0.05	1.00 ± 0.06	658.23 ± 2.40	1330 ± 0.01	90.7 ± 1.96
PKS + SS (90:10)	2.29 ± 0.13	0.60	0.65 ± 0.04	0.87 ± 0.04	713.51 ± 2.36	1380 ± 0.01	95.3 ± 1.86
PKS + SA (95:5)	2.33 ± 0.15	0.60	0.66 ± 0.04	0.91 ± 0.04	640.33 ± 5.68	1340 ± 0.008	87.0 ± 2.89
PKS + SA (90+10)	2.66 ± 0.11	0.60	0.75 ± 0.03	0.99 ± 0.02	710.10 ± 2.63	1360 ± 0.006	92.3 ± 0.10
PKS + SS + SA (90:5:5)	2.78 ± 0.08	0.60	0.79 ± 0.02	0.99 ± 0.03	593.61 ± 2.60	1300 ± 0.01	93.0 ± 0.49
PKS + SS + SA (80:10:10)	2.40 ± 0.13	0.60	0.68 ± 0.04	0.88 ± 0.04	553.00 ± 2.70	1270 ± 0.008	85.0 ± 0.73

**Table 3:** Proximate analysis from different ratios of binder with PKS pellets.

Proximate analysis (dry basis)					
	Moisturecontent	Volatile matter,	Fixed carbon,	Ash content	High heating
Pellet ratio	(wt%)	VM (wt%)	FC (wt%)	(wt%)	value,HHV (MJ/kg)^a^
Raw PKS (100:0)	3.37	65.41	13.47	17.75	14.82
PKS + SS (95:5)	3.42	77.53	16.58	2.47	17.93
PKS + SS (90:10)	3.93	84.14	10.75	1.18	16.91
PKS + SA (95:5)	2.92	76.36	17.76	2.96	18.16
PKS + SA (90+10)	3.43	80.29	14.72	1.56	17.71

a Calculated by using correlation proposed by Parikh et al., where HHV = 0.3536FC+0.1559VM−0.0078A [5].

**Fig. 4:**
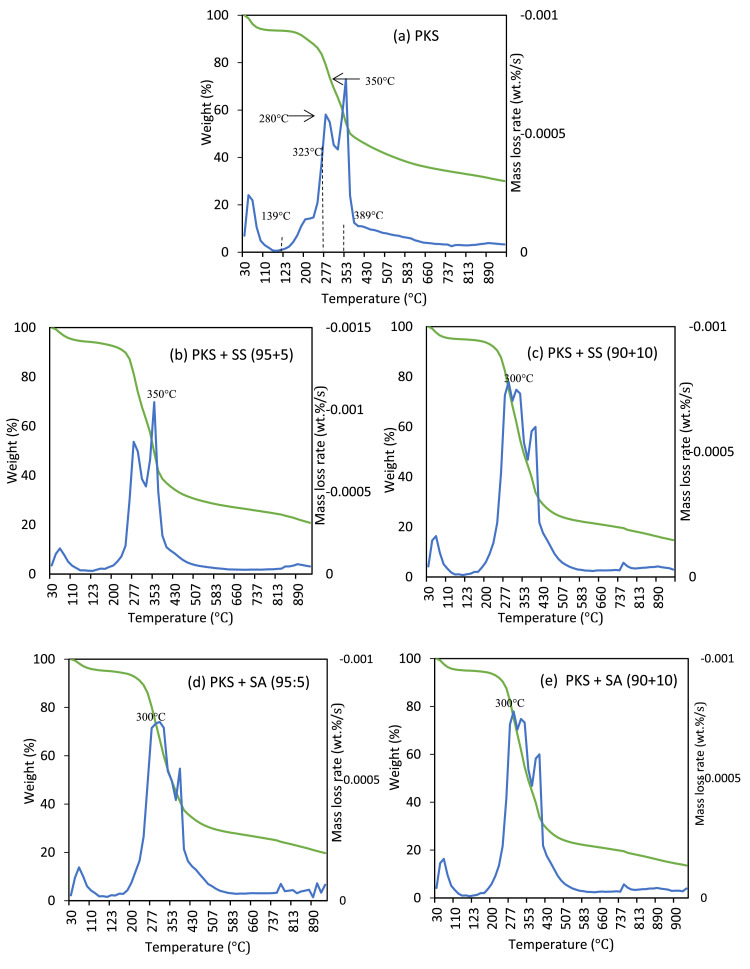
TG and derivative thermal gravimetric (DTG) curves of palm kernel shell pellet at different binder level of (a) raw PKS (100:0), (b) PKS + SS (95:5), (c) PKS + SS (90:10), (d) PKS + SA (95:5), (e) PKS + SA (90+10).

Thermo gravimetric analysis which includes thermal gravimetric (TG) and derivative thermal gravimetric (DTG) of the sample was performed to identify the materials’ thermal stability. This analysis also helped to assess the weight percentages of volatile matter, fixed carbon, moisture, and ash content in the pellets. The significance of both volatiles and fixed carbon is that they conveniently indicate which biomass can be ignited and subsequently gasified or oxidized, depending on how the biomass is to be applied as an energy source. Ash content, on the other hand, is inversely proportional to the available energy stored in the fuel; higher ash content leads to the proportionate reduction of the available energy of the fuel. High ash content also leads to slagging problems in the gasifier system and blockages of airways, especially when it reacts and forms sticky liquid. Nevertheless, a proper ash removal system can solve this problem. With respect to moisture content, it will reduce the calorific value of the solid fuel and also lower the product gas heating value. This is due to the energy loss in evaporating the excess moisture content of the biomass. The TG and DTG profile portraying the thermal decomposition characteristics are shown in Fig. 4. As observed in Fig. 4a, two shoulder peaks occurred in the DTG profile at around 280 °C and 350 °C. The peak observed at 139–323 °C was attributed to the decomposition of hemicellulose component [6]. The peak observed in the region of 323–389 °C was attributed to the composition of cellulose.

## 2 Experimental Design, Materials and Methods

### 2.1 Materials

PKS collected from Malaysian Palm Oil board (MPOB) were cleaned and dried at 105 °C for 24 h to prevent the formation of fungus. PKS were then crushed into fine powder of the size of 300 µm. SA (∼99%) and SS were blended with PKS at desired ratio.

### 2.2 Method

#### 2.2.1 Pelleting process

**Fig. 5:**
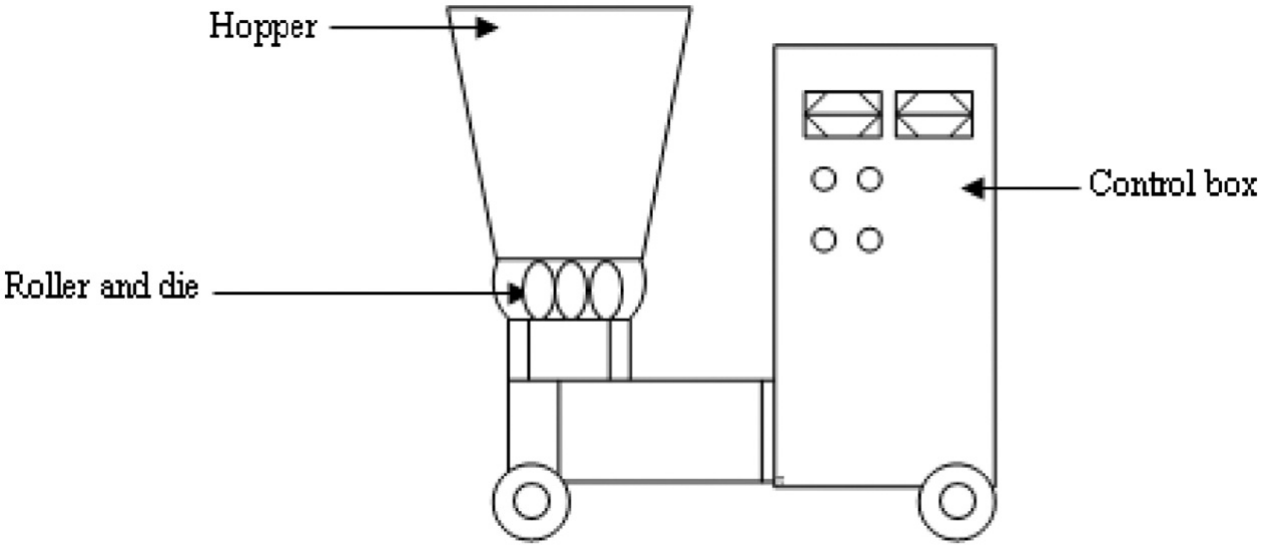
The schematic diagram of the pelletizer (Model WD229).

The process of producing pellets involves placing ground material under high pressure and temperature, and forcing it through several small hole (die).The sample prepared was compressed by a roller and the sample then fuses together to form a solid mass using a pellet mill (Model WD 229) at 100 °C and 50 MPa. Fig. 5 shows a schematic diagram of the pelletizer for better visualization/understanding. 1 kg of PKS and binder mixture was measured to the ratio summarized in Table 1. The mixture was blended and transferred to the pelleting machine for densification into diameter and length of 5 mm and 20–30 mm respectively.

#### 2.2.2 Physical properties of biomass raw material and binders

A proximate analysis of the PKS was done using a Mettler Toledo Thermogravimetric Analyzer which gives composition of a fuel in terms of moisture content, volatile matter, fixed carbon and ash; conducted according to ASTM Standard Test Method E870-82. Bulk density of the pellets produced was determined based on ASABE standard [7]. A measuring cylinder of 250 cm^3^ was used. The samples were poured slowly into the measuring cylindrical until it reached 250 cm^3^. The weight of the material with the measuring cylinder was measured and bulk density was calculated. The process was repeated for three times. Hardness test was conducted using Durometer. The resistance force was measured through the penetration of a pin into the material under a known spring load. The amount of penetration was converted to hardness reading on a scale of 100.

#### 2.2.3 Thermal decomposition behavior of the pellets

PKS pellets at different binder levels were selected to conduct the thermogravimetric analysis in investigating the thermal decomposition behavior. Samples were heated up to 105 °C at the rate of 10 °C/min and then up to 900 °C at the rate of 25 °C/min. Mass evolved at 105 °C was taken to be considered as moisture, while mass evolved between 105 °C and 900 °C consisted of fixed carbon and ash. Fixed carbon content was determined as the difference between 100% and the cumulated value of volatile matter and ash on a dry basis. Nitrogen and air were used as carrier gases at the flow rate of 100 mL/min. Experiments were carried out in triplicate and the relative error among the data of TGA was controlled to be less than 5%.

## Declaration of Competing Interest

The authors declare that they have no known competing financial interests or personal relationships that could have appeared to influence the work reported in this paper.

## Appendix Supplementary materials

Image, application 1
